# Pro-Inflammatory Cytokine Induction of 11β-hydroxysteroid Dehydrogenase Type 1 in A549 Cells Requires Phosphorylation of C/EBPβ at Thr235

**DOI:** 10.1371/journal.pone.0075874

**Published:** 2013-09-26

**Authors:** Cristina L. Esteves, Manu Verma, Ewa Róg-Zielińska, Val Kelly, Shuji Sai, Amandine Breton, Francesc X. Donadeu, Jonathan R. Seckl, Karen E. Chapman

**Affiliations:** 1 Endocrinology Unit, University/BHF Centre for Cardiovascular Science, Queen’s Medical Research Institute, The University of Edinburgh, Edinburgh, United Kingdom; 2 Division of Developmental Biology, The Roslin Institute, The University of Edinburgh, Edinburgh, United Kingdom; Fudan University, China

## Abstract

11β-hydroxysteroid dehydrogenase type 1 (11β-HSD1) converts inert glucocorticoids into active forms, thereby increasing intracellular glucocorticoid levels, important to restrain acute inflammation. 11β-HSD1 is induced by pro-inflammatory cytokines in a variety of cells. Here, we show 11β-HSD1 expression in human A549 epithelial cells is increased by pro-inflammatory cytokines (IL-1α/TNFα) via the P2 promoter of the *HSD11B1* gene. Inhibition of p38 MAPK attenuated the pro-inflammatory cytokine induction of mRNA encoding 11β-HSD1 as well as that encoding C/EBPβ. IL-1α/TNFα-induced phosphorylation of C/EBPβ at Thr235 was also attenuated by p38 MAPK inhibition suggesting involvement of a p38 MAPK-C/EBPβ pathway. siRNA-mediated knock-down of C/EBPβ and NF-κB/RelA implicated both transcription factors in the IL-1α/TNFα induction of *HSD11B1* mRNA. Transient transfections of *HSD11B1* promoter-reporter constructs identified the proximal region of the P2 promoter of *HSD11B1* as essential for this induction. IL-1α increased binding of C/EBPβ to the *HSD11B1* P2 promoter, but this was not observed for NF-κB/RelA, suggesting indirect regulation by NF-κB/RelA. Ectopic expression of mutant chicken C/EBPβ constructs unable to undergo phosphorylation at the threonine equivalent to Thr235 attenuated the IL-1α-induction of *HSD11B1*, whereas mimicking constitutive phosphorylation of Thr235 (by mutation to aspartate) increased basal expression of *HSD11B1* mRNA without affecting IL-1α-induced levels. These data clearly demonstrate a role for both C/EBPβ and NF-κB/RelA in the pro-inflammatory cytokine induction of *HSD11B1* in human epithelial cells and show that p38 MAPK-induced phosphorylation of C/EBPβ at Thr235 is critical in this.

## Introduction

11β-hydroxysteroid dehydrogenase type 1 (11β-HSD1) catalyses the conversion of the intrinsically inert glucocorticoids, cortisone and 11-dehydrocorticosterone, to active forms; cortisol and corticosterone, respectively, thus increasing intracellular glucocorticoid action [1]. Deficiency in, or inhibition of, 11β-HSD1 exacerbates acute inflammation although it is protective against the low-grade chronic inflammation associated with metabolic and cardiovascular disease [2,3]. 11β-HSD1 expression is increased at sites of inflammation, including in arthritis [4,5], obesity [6,7] and atherosclerosis [8], possibly mediated by the pro-inflammatory cytokines, IL-1α/β and TNFα, which increase levels of mRNA encoding 11β-HSD1 in a variety of cells [2]. The *HSD11B1* gene encoding 11β-HSD1 is transcribed from two different promoters, P1 and P2, which by RNA splicing produce the same protein [9]. Whilst activity of the P2 promoter is dependent on members of the CCAAT/enhancer binding protein (C/EBP) transcription factor family [9,10], the P1 promoter is C/EBP-independent [9]. Here we used A549 human lung epithelial cells, because unlike most cell lines, A549 cells express endogenous 11β-HSD1 and have previously been used as a cell model to demonstrate a requirement for C/EBPβ in glucocorticoid-regulation of the 11β-HSD1 promoter [11].

Previous work has associated IL-1 and TNFα induction of *HSD11B1* in human hepatoma cells with the p38 MAPK pathway and C/EBPβ [12] and in mouse mesenchymal stromal cells, with NF-κB signalling [13]. The latter is mediated via the P1 promoter. Raised levels of IL-1α/β and TNFα are a common feature of inflammation. These cytokines bind to cell surface receptors to activate, among others, the nuclear factor kappa B (NF-κB) and mitogen-activated protein kinase (MAPK) signalling pathways [14–17]. Key downstream mediators/effectors of inflammatory signalling include NF-κB [18] and the C/EBP members, C/EBPβ and C/EBPδ [19–21]. Two main isoforms of C/EBPβ are produced from *CEBPB* mRNA; the liver-enriched activator protein (LAP), and the liver-enriched inhibitor protein (LIP) which acts as inhibitor of transcription (although in some cases it may also act as co-activator [22]). The NF-κB family of transcription factors encompasses five proteins: RelA (p65), RelB, c-Rel, p50 and p52, with RelA being typically the active component of NF-κB activation complexes [18].

Here, we have investigated the involvement of p38 MAPK, C/EBPβ and NF-κB pathway in the pro-inflammatory induction of *HSD11B1* in the cytokine responsive human lung A549 epithelial cell line [11].

## Materials and Methods

### Cell culture

Human lung epithelial A549 cells were maintained in Dulbecco’s Modified Eagle’s Medium (DMEM; Lonza, Tewkesbury, UK), supplemented with 10% fetal bovine serum (Lonza), 100U/ml penicillin and 100µg/ml streptomycin as previously described [11]. CCRF-CEM human leukemia cells were cultured in RPMI 1640 (Lonza) supplemented with 10% fetal bovine serum (Lonza), 100U/ml penicillin and 100µg/ml streptomycin as described [23].

Unless stated otherwise, A549 cells were treated with 10ng/ml IL-1α (R&D Systems, MA, USA) or 20ng/ml TNFα (R&D Systems) for 24h at 37°C in serum-free DMEM medium supplemented with 100U/ml penicillin and 100µg/ml streptomycin. In the experiments designed to inhibit MAPK pathways, inhibitors of p38 (10µM SB203580; Invitrogen, Paisley, UK), c-Jun N-terminal kinase II (JNK II; 20µM SP600125; Calbiochem, Darmstadt, Germany) or mitogen-activated protein kinase/extracellular signal-regulated kinase 1/2 (MEK1/2; 0.5µM PD0325901; Calbiochem) were added 1h prior to treatment with IL-1α. Inhibitors were all dissolved in DMSO and diluted in serum-free DMEM for addition to cells (final concentration of DMSO was ≤0.1%). For RNA analysis, cells were collected 24h following addition of IL-1α. For protein analysis, cells were collected at various times ranging from 15min to 6h. Cycloheximide (CHX, 0.1µM), which was used to inhibit *de novo* protein synthesis, was added to cells 1h prior to IL-1α/TNFα treatment for 4h.

### RNA extraction and analysis

A549 cells were harvested in Trizol (Invitrogen) and RNA extracted as described [11]. RNA (1µg) was reversed transcribed using SuperScript III (Invitrogen) as described [24] and amplified by non-quantitative PCR or by real-time quantitative (q)PCR using a Roche LightCycler 480. In the assays designed to test the usage of the *HSD11B1* P1 and P2 promoters, non-quantitative PCR was performed using the forward primers: pP1, 5’-GAAGTCAGATTTGTTCGAAATCTTG-3’; pP2, 5’-GGAGGTTGTAGAAAGCTCTG-3’ or pC, 5’-TTCTGCAAACGAGGAATTCAG-3’ to amplify transcripts from the P1 and P2 promoters, or a region common to both transcripts, respectively. The common reverse primer was 5’GTAGAGTTTCTTTTGACCTCG- 3’. PCR was carried out at 96°C for 5min, followed by 35 cycles at 96°C for 30s and 56°C for 1min.

For qPCR, primer-probe sets were purchased from Applied Biosystems (Warrington, UK): 11β-HSD1 (Hs00194153_m1), C/EBPβ (Hs00270923_s1), NF-κB/RelA (Hs00153294_m1). TATA binding protein was used as an internal control (TBP; Hs00427620_m1).

### IL-8 ELISA

IL-8 was measured using the Human IL-8 ELISA development kit (Peprotech, Rocky Hill, NJ, USA) according to the manufacturers’ instructions. Briefly, following overnight incubation with capture antibody and blocking with 1% bovine serum albumin for 1h, samples and standards (human recombinant IL-8) were added to the wells, incubated with detection antibody and then with avidin-HRP conjugate. ABTS Liquid Substrate (Sigma, Dorset, UK) was used for detection.

### Transfections

For siRNA transfections, A549 cells were seeded at a density of 7.5x10^4^ cells per well of a 12-well plate and transfected the following day with 80pmol siRNA using Lipofectamine 2000 (Invitrogen) according to the manufacturer’s instructions. 24h after transfection, cells were harvested for protein analysis or further incubated with pro-inflammatory cytokines for 24h prior to RNA extraction. siRNAs (Applied Biosystems) comprised scrambled RNA (AM4611 negative control 1), C/EBPβ (ID114495) or NF-κB/RelA (ID s11914).

For plasmid transfections, A549 cells were transfected as previously described [11]. Briefly, 1.5×10^5^ cells were seeded per well of a 6-well plate and transfected the next day using Lipofectamine 2000 (Invitrogen) with 250ng reporter plasmid and 250ng pRSV-*LacZ* (as internal control) or, for transfections with C/EBPβ mutants, with 50ng of expression plasmid encoding wild-type chicken C/EBPβ or C/EBPβ in which Thr220 (equivalent to Thr 235 in human C/EBPβ) has been mutated to alanine (T220A) or aspartate (T220D) [25,26] (a gift from E. Kowenz-Leutz and A. Leutz, Max Delbrüeck Centre for Molecular Medicine, Berlin). Cells were treated the following day with IL-1α and harvested 24h later. Reporter plasmids have been described previously [10] and comprise a 5′ deletion series of the human [27] or rat [10] *HSD11B1* P2 promoter, designated H11β1 or R11β1 (5′-endpoint/3’-endpoint) for human and rat, respectively; an internal deletion series of R11β1(−1799/+49), designated RΔ11β1(5′-endpoint of deletion/3’-endpoint of deletion) and derivatives of R11β1(−196/+49) with mutations in footprint (FP)3, FP4, or both FP3 and FP4 [10].

### Western blot analysis

Cells were harvested directly into lysis buffer (0.125M Tris-HCL pH 6.8, 2% sodium dodecyl sulphate, 10% glycerol) in the presence of a protease inhibitor cocktail (P2714; Sigma-Aldrich, Dorset, UK) and heated at 100°C. Electrophoresis was carried out on 4-12% NuPage Bis-Tris gels (Invitrogen). After transfer, blots were probed with antibodies to detect C/EBPβ (1:500 dilution, Santa Cruz Biotechnology, Santa Cruz, CA), Thr235 phosphorylated C/EBPβ (1:1000 dilution, Cell Signaling, Danvers, MA), NF-κB/RelA (1:500 dilution, Santa Cruz Biotechnology, Santa Cruz, CA) or β-tubulin, used as an internal control (1:10000 dilution, Chemicon/Millipore, Watford, UK). Secondary antibodies were anti-rabbit IgG-HRP (1:2000 dilution, Santa Cruz Biotechnology Inc.), anti-mouse IgG-HRP (1:4000 dilution, Cell Signaling, Danvers, MA) or anti-rabbit IgG IRDye 800CW (dilution 1:10000; LI-COR Biosciences, Cambridge, UK). The resulting bands were visualised on chemiluminescent films (GE Healthcare) using the ECL system or using the Odyssey Infrared Imaging System (LI-COR Biosciences), as appropriate.

### Immunocytochemistry

Cells were grown on coverslips in 12 well plates, treated with IL-1α and TNFα, fixed with 4% paraformaldehyde (Sigma-Aldrich) and then incubated for 16h at 4°C with antibodies against C/EBPβ or NF-κB/RelA (1:200 dilution, Santa Cruz Biotechnology) followed by anti-rabbit AlexaFluor 568 secondary antibody (Invitrogen). Cells were then stained with DAPI (Sigma-Aldrich) for 10min. Coverslips were mounted using fluoroshield (Sigma) and visualised on an Axiovert 25 fluorescence microscope.

### Chromatin immunoprecipitation (ChIP) assays

ChIP assays were carried out using an Upstate EZ ChIP kit (Millipore, Billerica, MA) as previously described [11] or following the protocol from Dahl and Collas [28] with adaptations. Briefly, A549 cells were grown to confluence in 10cm dishes, then treated with IL-1α for 24h. Proteins were cross-linked to DNA by addition of 1% paraformaldehyde then cells lysed in 50mM Tris-HCl pH 8.0, 1% sodium dodecyl sulphate, 10mM EDTA. Chromatin was sheared by sonication using a Soniprep 150 (MSE; Beckenham, Kent, UK) with 10 x 10s pulses (10 amplitude microns) keeping cells on ice between pulses. Chromatin aliquots were immunoprecipitated using Dynabeads-protein A (Invitrogen) with C/EBPβ, NF-κB/RelA or control IgG antibody (Santa Cruz). DNA was amplified by qPCR using primers (5'-AGTCCTGTACAGTCATGAGCTTGG-3' and 5'-ATTTCCCTGTCAGAGCAGCGATTG-3') to amplify the region containing the FP3 and FP4 C/EBP binding sites of the *HSD11B1* promoter [10,11] and putative NF-κB binding region (see Results for details). The primers (5'-AGGAAGTGTGATGACTCAGGTTT-3' and 5'-CTCCGGTGGCTTTTTATATCATC-3') were used to amplify the NF-κB/RelA binding site in the *IL-8* promoter. Data are expressed relative to levels of input DNA in immunoprecipitations.

### Statistical analysis

Data were analyzed by Student’s *t* test or by one- or two-way ANOVA followed by *post hoc* Tukey tests using SigmaStat 2.03 statistical software. Significance was set at p<0.05.

## Results

### Pro-inflammatory cytokines increase *HSD11B1* mRNA levels in A549 cells via the P2 promoter

**Figure 1:**
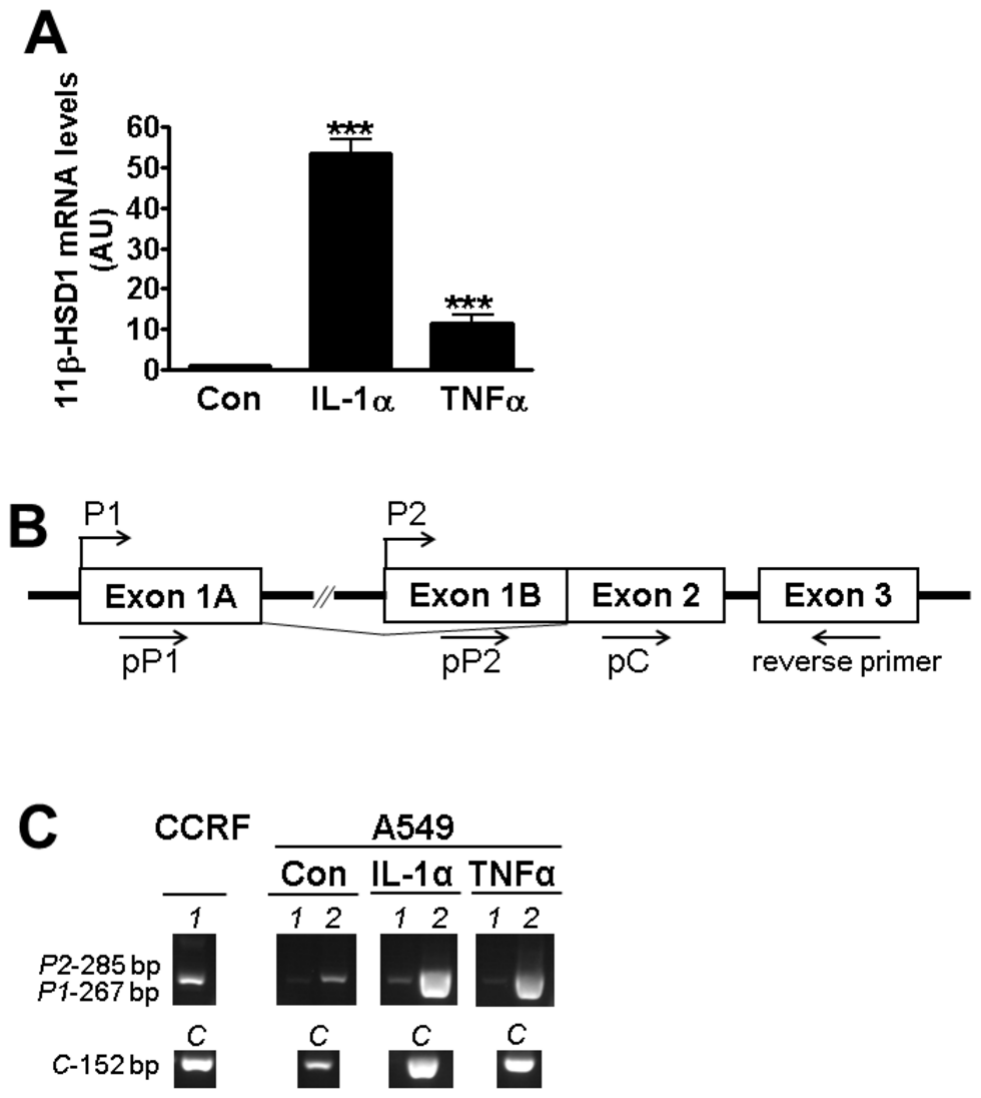
Pro-inflammatory cytokines increase levels of *HSD11B1* mRNA from the P2 promoter in A549 cells. (**A**) qPCR measurement of *HSD11B1* mRNA following 24h treatment of A549 cells with IL-1α (10ng/ml) or TNFα (20ng/ml) (black bars). Controls (white bars) were untreated. All values are expressed relative to levels in untreated cells, arbitrarily set to 1 and are mean ± SEM; n≥6. *, significantly different from control. ***, p<0.0001. (**B**) Schematic representation of the *HSD11B1* gene showing exons 1A, 1B, 2 and 3 (open boxes) and the corresponding start of transcription for the P1 and P2 promoters (bent arrows). The forward primers used to amplify transcripts from P1 (pP1), P2 (pP2) or a region common to transcripts originating from both P1 and P2 promoters (pC) are indicated. The reverse primer was used for all PCR reactions. (**C**) Gel showing PCR products from transcripts initiating at P1 (*1*, 267bp), P2 (*2*, 285bp) and a region common to transcripts originating from either promoter (*C*; 152bp) produced using cDNA from untreated (Con) A549 cells or following 24h treatment with IL-1α or TNFα. CCRF-CEM human leukaemia cells, which express *HSD11B1* predominantly from the P1 promoter, served as a positive control for P1-initiated transcripts. All PCR products were run together and in the same gel.

Treatment of A549 cells with IL-1α or TNFα robustly increased levels of mRNA encoding 11β-HSD1 (Figure 1A). Concomitantly, IL-8 secretion was increased confirming that A549 cells are cytokine-responsive (data not shown). The gene encoding 11β-HSD1 is transcribed from one of two promoters, P1 or P2, which by RNA splicing produce the same protein [9]. Non-quantitative PCR amplification of *HSD11B1* mRNA using primers specific for transcripts originating from promoter P1 or P2, or a region common to both transcripts (Figure 1B), showed that pro-inflammatory cytokine induction of *HSD11B1* in A549 cells is mediated by the P2 promoter (Figure 1C).

### p38 MAPK and C/EBPβ phosphorylation at Thr235 are implicated in the cytokine-induction of *HSD11B1* mRNA

**Figure 2:**
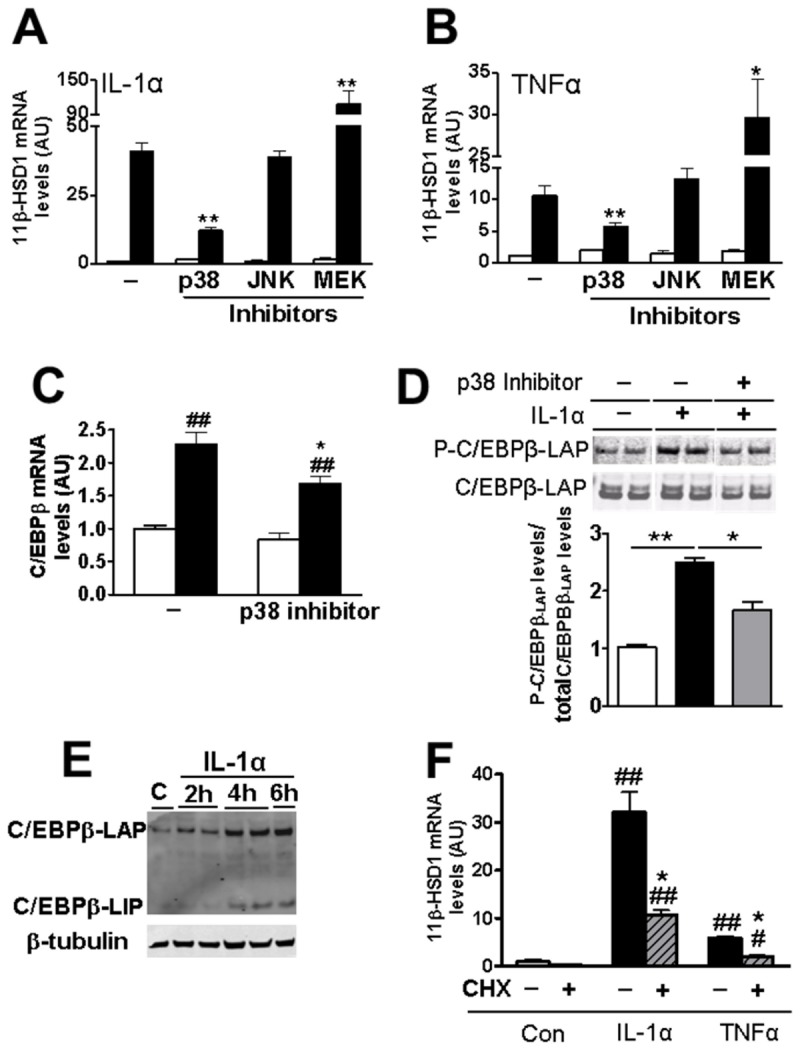
Inhibition of p38 MAPK attenuates IL-1α induction of mRNAs encoding 11β-HSD1 and C/EBPβ and prevents IL-1α induced C/EBPβ phosphorylation. (**A**, **B**) qPCR measurement of levels of mRNA encoding 11β-HSD1 in A549 cells without inhibitor (-) or pre-incubated for 1h with inhibitors of p38 MAPK (p38, SB203580; 10µM), JNK II (SP600125; 20µM) or MEK I (PD0325901; 0.5µM) prior to 24h treatment with IL-1α (**A**) or TNFα (**B**) (black bars). White bars; no cytokine treatment. All data are expressed relative to levels in untreated cells (no inhibitor, no cytokine), arbitrarily set to 1, and are mean ± SEM; n≥3. *, significantly different from IL-1α-stimulated control. *, p<0.05; **p<0.001. (**C**) qPCR measurements of levels of mRNA encoding C/EBPβ in cells treated for 24h with IL-1α with (p38 inhibitor), or without (-) prior addition of p38 MAPK inhibitor. Data are expressed relative to levels in untreated control cells (no inhibitor, no cytokine), arbitrarily set to 1, and are mean ± SEM; n≥6. *, significantly different from IL-1α-stimulated control. *, p<0.05. #, significantly different from unstimulated cells. ##, p<0.001. (**D**) Western blot showing levels of phosphorylated C/EBPβ-LAP (P-C/EBPβ-LAP); upper image) in untreated A549 cells (-p38 MAPK, -IL-1α; white bar), or following 15min treatment with IL-1α alone (-p38 MAPK, +IL-1α; black bar) or with prior addition of p38 MAPK inhibitor for 1h (+p38 MAPK, +IL-1α; grey bar; SB203580, 50µM). The blot (20µg protein/lane) was stripped and reprobed with C/EBPβ antibody (bottom membrane) to detect total C/EBPβ-LAP. Protein bands were quantified using the Odyssey Infrared Imaging System and the ratio of P-C/EBPβ-LAP/total C/EBPβ-LAP calculated. Data are expressed relative to levels in untreated cells (no inhibitor, no cytokine), arbitrarily set to 1, and are mean ± SEM; n=3. *, p<0.05; **, p<0.001. (**E**) Western blot showing increased levels of C/EBPβ-LAP and C/EBPβ-LIP isoforms after IL-1α treatment for up to 6h, compared to control untreated cells (C). (**F**) qPCR measurement of levels of mRNA encoding 11β-HSD1 in A549 cells incubated for 1h with CHX (+) or without CHX (-) followed by IL-1α/TNFα treatment for 4h or control (Con). Data are expressed relative to levels in untreated cells (no CHX, no cytokine), arbitrarily set to 1, and are mean ± SEM; n=3. *, p<0.05 significant effect of CHX. #, significantly different to untreated cells (no CHX, no cytokine). #, p<0.05; # #, p<0.001.

**Figure 3:**
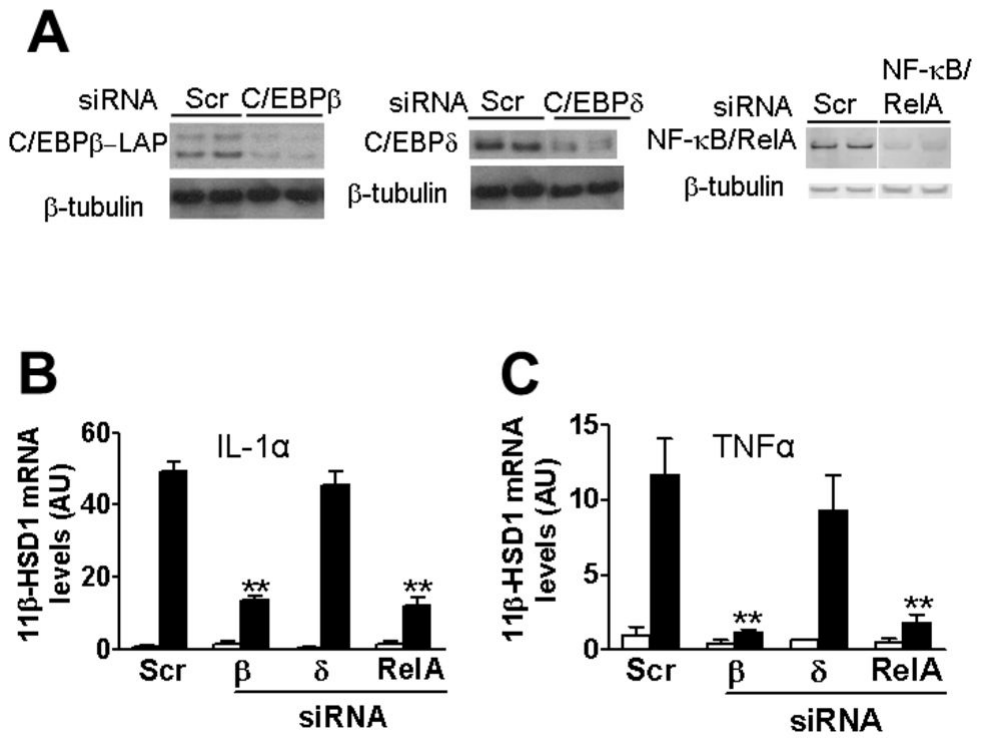
siRNA mediated knock-down of C/EBPβ or NF-κB/RelA attenuates the pro-inflammatory cytokine induction of *HSD11B1*. (**A**) Representative western blots (12.5µg protein/lane) showing levels of C/EBPβ (left panel), C/EBPδ (middle panel) and NF-κB/RelA (right panel) 24h after transfection of cells with scrambled RNA (Scr; as control) or siRNAs targeting C/EBPβ, C/EBPδ or NF-κB/RelA. Blots were stripped and reprobed with β-tubulin antibody, as loading control. In the C/EBPδ and NF-κB/RelA westerns, all samples were analysed in the same gel but not all in adjacent lanes. (**B**, **C**) Real-time PCR measurement of *HSD11B1* mRNA in untreated cells (white bars) or cells treated for 24h with IL-1α (**B**; black bars) or TNFα (**C**; black bars), 24h after transfection with scrambled RNA (Scr) or siRNA targeting C/EBPβ (β), C/EBPδ (δ) or NF-κB/RelA (RelA). Data are expressed relative to levels in untreated cells transfected with scrambled RNA, arbitrarily set to 1. Values, in arbitrary units (AU), are mean ± SEM; n≥5. **, p<0.001, compared to cells transfected with scrambled RNA followed by IL-1α/TNFα treatment.

Pro-inflammatory cytokines activate MAPK signalling (p38, JNK and MEK/ERK). Inhibition of p38 MAPK attenuated the IL-1α/TNFα induction of *HSD11B1* (Figure 2A, B) whereas inhibition of JNKII had no effect. MEK1/2 inhibition increased *HSD11B1* mRNA above levels seen with IL-1α/TNFα alone (Figure 3A, B) without affecting *CEBPB* mRNA levels (2.27±0.19 vs 2.03±0.09 arbitrary units, for cells without and with MEK1/2 inhibitor, respectively). None of the inhibitors affected basal *HSD11B1* mRNA levels, in the absence of IL-1α. IL-1α treatment of A549 cells increased levels of C/EBPβ mRNA (Figures 2C; 24h time point) and levels of both C/EBPβ-LAP and C/EBPβ-LIP protein isoforms (time course 2 to 6h), though levels of C/EBPβ-LAP, the activator isoform accumulated earlier and to a greater extent than the inhibitory C/EBPβ-LIP isoform (Figure 2E). CHX incubation of A549 cells prior to IL-1α/TNFα treatment for 4h attenuated the induction of *HSD11B1* mRNA levels indicating that pro-inflammatory cytokine induction of *HSD11B1* partially depends on *de novo* protein synthesis (Figure 2F). Moreover, IL-1α treatment of A549 cells for 15min resulted in the rapid phosphorylation of C/EBPβ on Thr235 (Figure 2D). Both the increase in *CEBPB* mRNA 24h after addition of IL-1α and the rapid increase in phosphorylated C/EBPβ-LAP were attenuated by p38 MAPK inhibition (Figure 2C, D), suggesting that p38 MAPK-mediated phosphorylation of C/EBPβ is involved in the cytokine induction of 11β-HSD1 and that this is sustained through increased C/EBPβ expression. Consistent with this idea, siRNA-mediated knock-down of C/EBPβ, which did not change basal *HSD11B1* mRNA levels, attenuated IL-1α/TNFα-induction of *HSD11B1* (Figure 3A-C). In contrast, although IL-1α/TNFα treatment increased levels of *CEBPD* mRNA encoding the related C/EBPδ transcription factor (1.45±0.13 and 1.49±0.17 following treatment with IL-1α and TNFα, respectively, relative to untreated cells, 1.00±0.10 arbitrary units; p<0.001), siRNA-mediated knock-down of C/EBPδ had no effect on the cytokine induction of *HSD11B1* (Figure 3). *CEBPA* mRNA levels were unaffected by cytokine treatment of A549 cells (0.54±0.21 and 0.79±0.10 following treatment with IL-1α and TNFα, respectively, relative to untreated cells, 1.00±0.18 arbitrary units). Overall these results confirm that C/EBPβ is required for the full cytokine induction of *HSD11B1* in A549 epithelial cells.

### siRNA-mediated knock-down of NF-κB/RelA attenuates *HSD11B1* induction by IL-1α/TNFα

NF-κB/RelA is required for the pro-inflammatory cytokine induction of mRNA encoding 11β-HSD1 in murine mesenchymal cells [13]. However, a role in epithelial cells has not been demonstrated. In human A549 cells, *RELA* mRNA levels were increased following treatment with IL-1α or TNFα (data not shown). siRNA was used to test whether NF-κB/RelA also plays a role in pro-inflammatory cytokine induction of *HSD11B1* in human A549 epithelial cells. Knock-down of NF-κB/RelA (Figure 3A) did not change basal *HSD11B1* mRNA levels but attenuated the induction of *HSD11B1* mRNA by IL-1α/TNFα (Figure 3B, C), demonstrating a role for the NF-κB/RelA pathway in the cytokine induction of *HSD11B1*.

### C/EBPβ and NF-κB/RelA locate to the nucleus in cytokine-treated A549 cells

**Figure 4:**
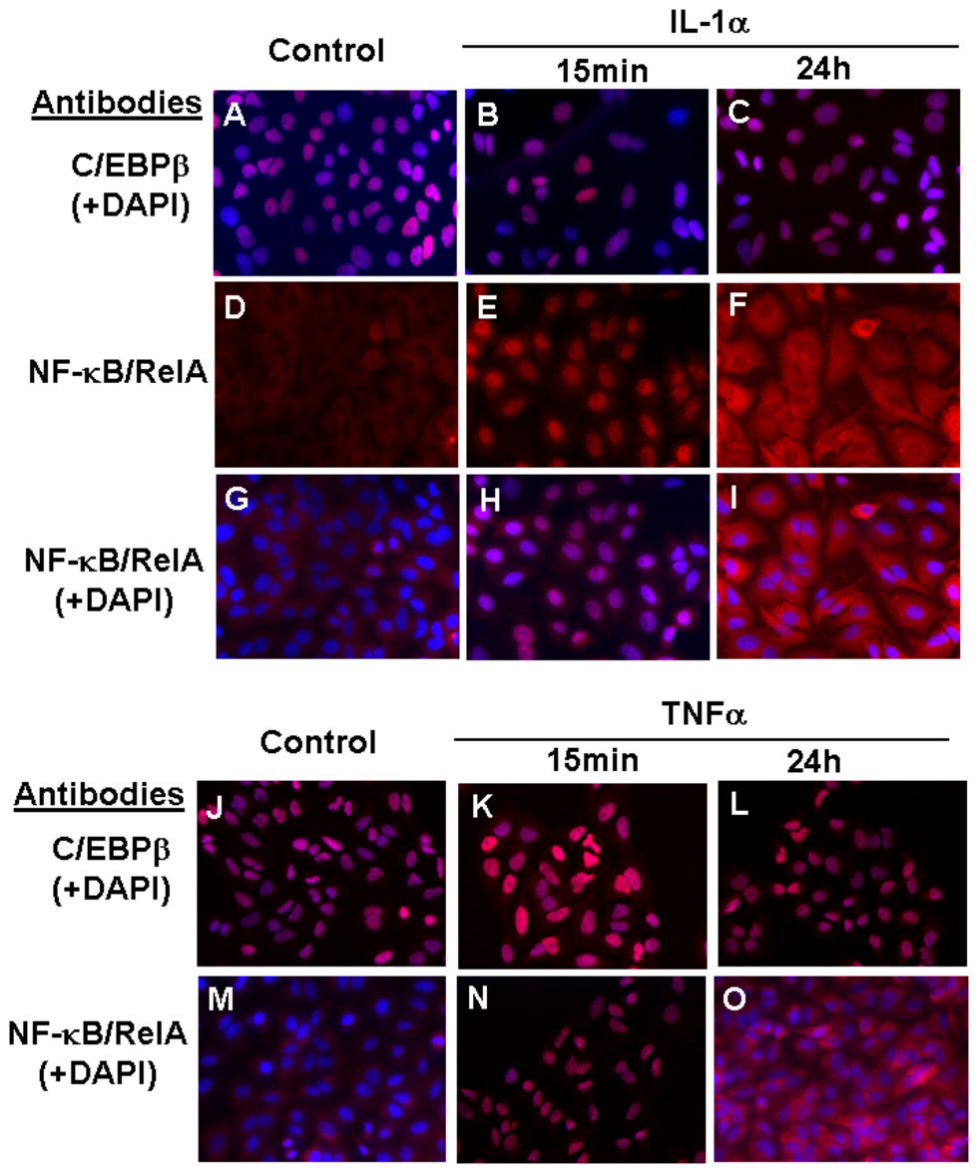
C/EBPβ and NF-κB/RelA are located in the nucleus in IL-1α/TNFα treated A549 cells. C/EBPβ is located in the nucleus in untreated and cytokine-treated A549 cells whereas NF-κB/RelA rapidly translocates to the nucleus in response to cytokine treatment. Micrographs show A549 cells 15min or 24h after addition of IL-1α (upper panels) or TNFα (lower panels), compared to untreated (control) cells. Red fluorescence shows immunodetection of C/EBPβ (A-C and J-L) or NF-κB/RelA (D-I and M-O). DAPI (blue) was used to stain nuclei. All micrographs were obtained with an objective magnification of x40.

Immunofluorescent staining of A549 cells showed nuclear localisation of C/EBPβ irrespective of cytokine treatment (Figure 4A-C, J-L). In agreement with the literature, NF-κB/RelA was restricted to the cytoplasm in untreated A549 cells (Figure 4D, G, M) but rapidly translocated to the nucleus following addition of IL-1α or TNFα (Figure 4E, H, N), largely returning to the cytoplasm 24h after treatment (Figure 4F, I, O).

### Pro-inflammatory cytokine induction of the P2 promoter of *HSD11B1* requires the C/EBPβ binding sites, FP3 and FP4

**Figure 5:**
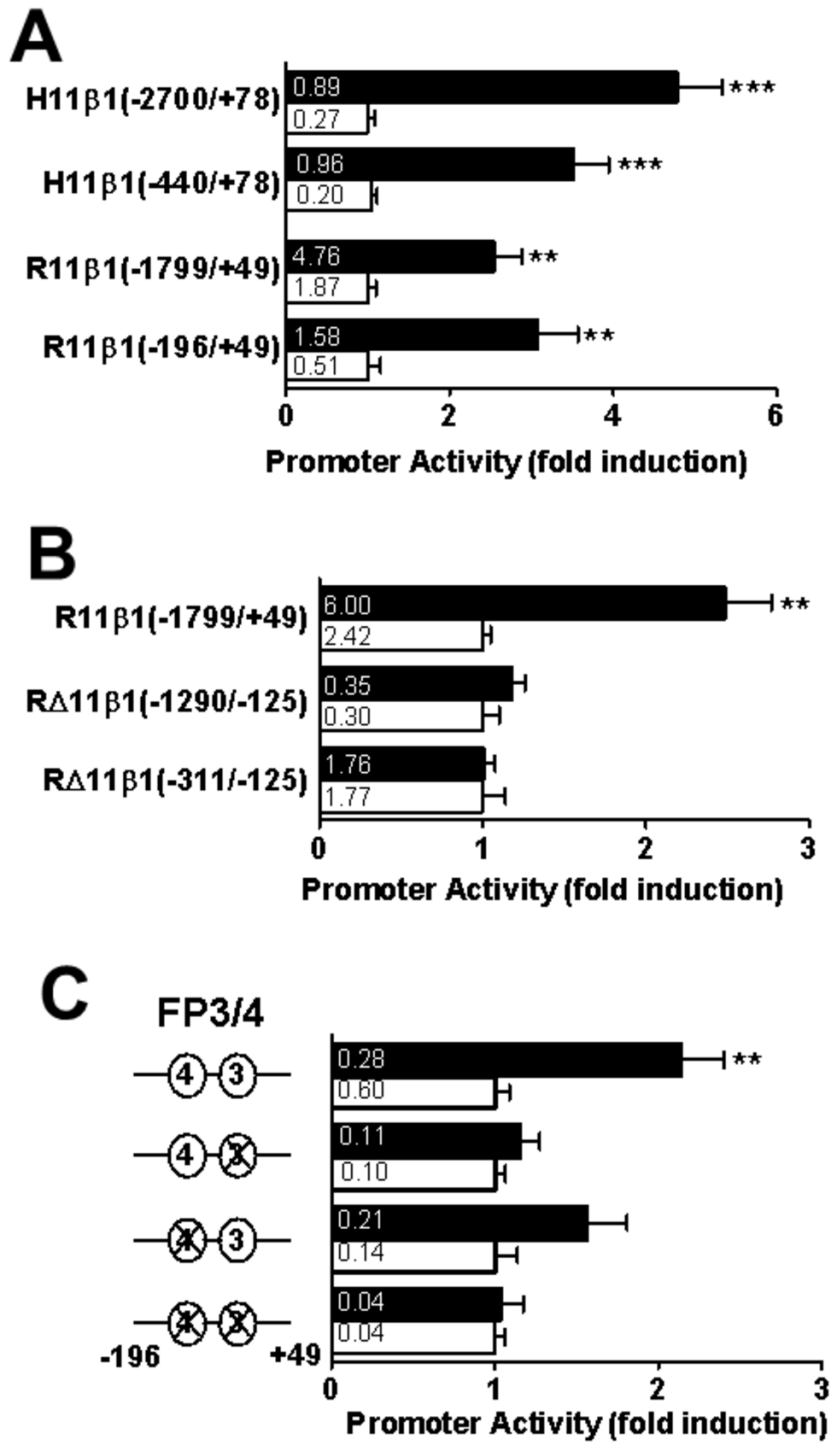
The proximal region is required for pro-inflammatory cytokine induction of the P2 promoter of *HSD11B1*. (**A**) Transiently transfected A549 cells showing IL-1α-induction of luciferase reporter constructs encoding the human *HSD11B1* P2 promoter; H11β1(-2700/+78) and H11β1(-440/+78), and the rat *Hsd11b1* P2 promoter; R11β1(-1799/+49) and R11β1(-196/+49). (**B**) Internal deletions of the rat *Hsd11b1* P2 promoter (-1799/+49) that removed -1290 to -125 R∆11β1(-1290/-125) or -311 to -125 R∆11β1(-311/-125) abolished IL-1α induction of promoter activity. (**C**) Mutation of FP3 and/or FP4 within the proximal promoter (-196/+49) of the rat *Hsd11b1* promoter abolished induction by IL-1α. Black bars, IL-1α-treated cells; white bars, untreated. Promoter activity is expressed as luciferase/β-galactosidase (internal control) activity. Values are fold induction relative to untreated cells transfected with the same plasmid (means ± SEM; n≥6). Absolute luciferase/β-galactosidase values (without normalization) are shown in the bars of the graph. *, significantly different from untreated cells transfected with the same plasmid. **, p<0.001; ***, p<0.0001.

To localise the region of the *HSD11B1* P2 promoter responsible for pro-inflammatory cytokine induction of 11β-HSD1, A549 cells were transfected with luciferase reporter constructs encoding regions of the human or rat *HSD11B1* gene. IL-1α treatment induced promoter-reporter constructs encoding -2700 to +78 of the human *HSD11B1* P2 promoter (the start of transcription is designated +1) or -1799 to +49 of the rat *Hsd11b1* promoter (Figure 5A). Promoter activity was unaffected by 5’-deletion of the human *HSD11B1* promoter to -440 or of the rat *Hsd11b1* promoter to -146, positioning the cytokine responsive region in the proximal region of the promoter. Furthermore, deletion of the region between -311 and -125 of the rat promoter abolished cytokine induction (Figure 5B), localising an essential region between -196 and -125 (though this could extend further 3’). This region, highly conserved between the rat and human *HSD11B1* genes, encompasses the C/EBP binding sites FP3 and FP4, which are critical for *HSD11B1* regulation by a variety of stimuli in a variety of cell types [10,11,24,29–31]. Mutation of FP3 or FP4 alone or in combination abolished induction of the promoter by IL-1α (Figure 5C), showing that both sites are necessary for cytokine induction of 11β-HSD1 and implicating C/EBPβ as a key mediator of the response.

### IL-1α increases binding of C/EBPβ, but not NF-κB/RelA, to the region encompassing FP3 and 4 in the P2 promoter of *HSD11B1*

**Figure 6:**
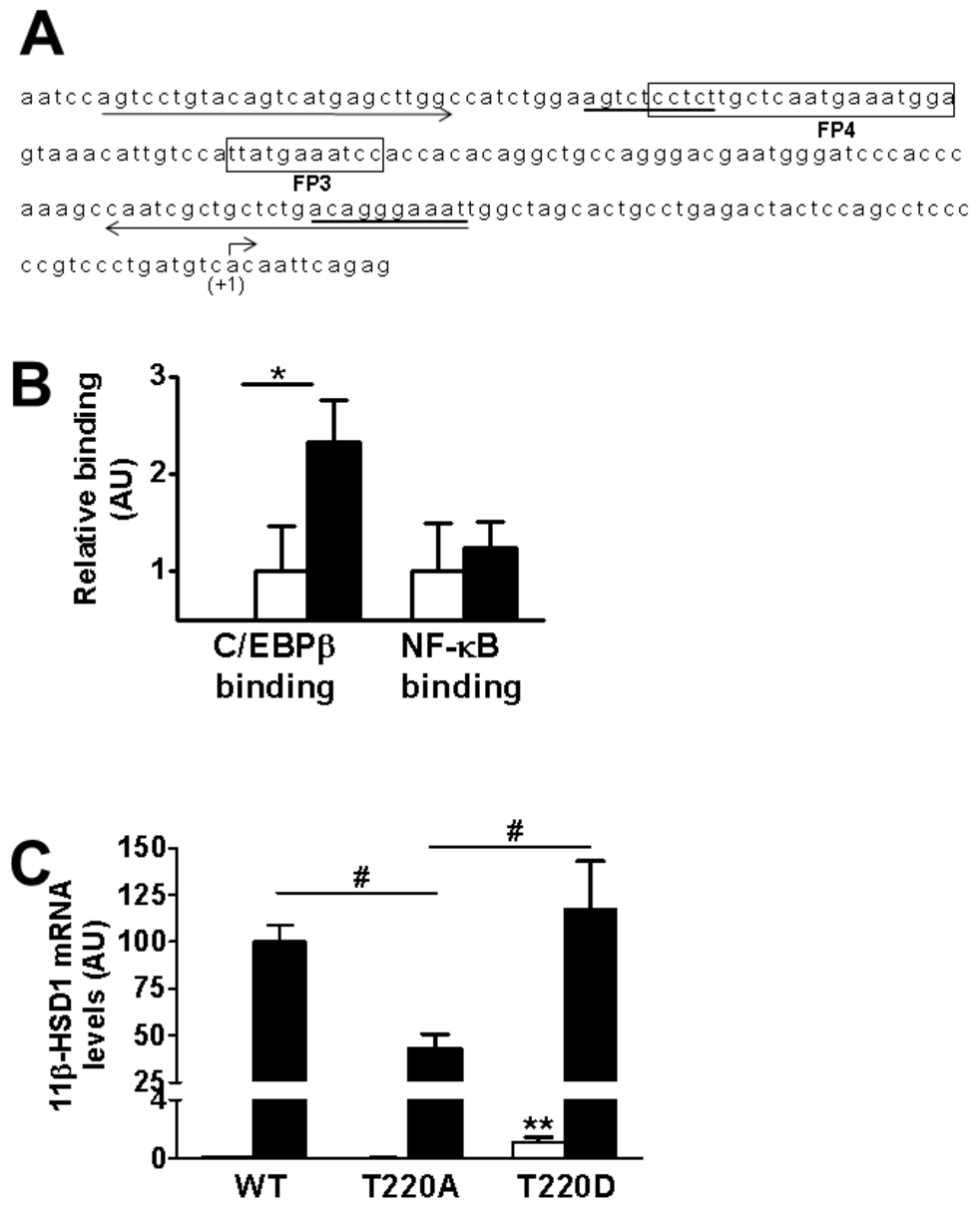
Chromatin immunoprecipitation assays show binding of C/EBPβ, but not of NF-κB/RelA, to the *HSD11B1* P2 promoter following IL-1α treatment. (**A**) Sequence of the region of the P2 promoter of the *HSD11B1* gene that is essential for induction by IL-1α, which includes the previously described C/EBP binding sites, FP3 and FP4 [10] (boxed). The transcription start site (+1) is indicated by a bent arrow. Putative NF-κB binding sites (predicted using AliBaba 2.1 software; Biobase, Biological databases) are underlined. Primers used in ChIP assays are indicated by arrows under the sequence. (**B**) qPCR quantification of ChIP assays showed C/EBPβ but not NF-κB binding to the P2 promoter of *HSD11B1* in cells treated with IL-1α (black bars). White bars, untreated cells. Values, in arbitrary units (AU), are mean ± SEM; n=5. *, p<0.05. (**C**) qPCR measurements of levels of mRNA encoding 11β-HSD1 in untreated (white bars) or following IL-1α treatment (black bars) of A549 cells transiently transfected with plasmids (50ng) encoding wild-type (WT) chicken C/EBPβ or mutants C/EBPβ(T220A) or C/EBPβ(T220D), abolishing or mimicking, respectively, constitutive phosphorylation of T220 (equivalent to T235 in human C/EBPβ). Data, in arbitrary units (AU), are expressed relative to levels in IL-1α-treated cells transfected with WT C/EBPβ, arbitrarily set to 100 and are mean ± SEM; n=6. **, p<0.001 compared to untreated cells transfected with WT C/EBPβ; #, p<0.05 compared to cells transfected with C/EBPβ(T220A) and treated with IL-1α.

The above experiments show that FP3 and FP4, known C/EBPβ binding sites in the *HSD11B1* promoter (Figure 6A), are required for pro-inflammatory cytokine induction of *HSD11B1*. As predicted, C/EBPβ binding to the FP3/FP4 region of the *HSD11B1* promoter was increased in IL-1α (Figure 6A, B) and in TNFα-treated cells (data not shown). In contrast, although IL-1α caused NF-κB/RelA to bind to the promoter of the gene encoding IL-8 in A549 cells (data not shown), no binding to the *HSD11B1* promoter was observed (Figure 6B), despite the prediction of putative binding sites for NF-κB in this region (Figure 6A).

### C/EBPβ phosphorylation at Thr235 is implicated in cytokine-induction of *HSD11B1* transcription

To test the role of C/EBPβ phosphorylation in the cytokine regulation of *HSD11B1*, A549 cells were transfected with expression plasmids encoding the chicken homologue of C/EBPβ or mutants in which Thr220 (equivalent to Thr235 in human C/EBPβ) was changed to alanine (C/EBPβ-T220A, abolishing phosphorylation at this key residue) or to aspartate (C/EBPβ-T220D, mimicking constitutive phosphorylation) [25,26]. Compared to the wild-type (WT) C/EBPβ, transfection with C/EBPβ-T220A attenuated cytokine induction of *HSD11B1* whereas C/EBPβ-T220D had no effect on *HSD11B1* induction, but increased basal *HSD11B1* mRNA levels in vehicle treated cells (Figure 6C).

## Discussion

We have previously shown that the P2 promoter is active in A549 epithelial cells, with little or no P1 activity [9], consistent with data from others showing the P2 promoter predominates in untreated A549 cells [32]. As in mesenchymal cells [13], pro-inflammatory cytokines increase activity of the C/EBP-dependent P2 promoter of *HSD11B1* [9] in A549 cells and this is associated with increased binding of C/EBPβ to the FP3/FP4 region of the promoter, similar to TNFα induction of *HSD11B1* in human HepG2 hepatoma cells [12]. Here, we also found NF-κB/RelA is required for IL-1α induction of the P2 promoter of *HSD11B1* in A549 epithelial cells, as it is in murine mesenchymal cells [13], though a direct interaction of NF-κB/RelA with the *HSD11B1* promoter in the latter was not tested. Our data show that the requirement for NF-κB/RelA for pro-inflammatory cytokine induction of *HSD11B1* in human epithelial cells does not involve direct binding to the P2 promoter.

Previous data have implicated p38 MAPK in the pro-inflammatory cytokine induction of 11β-HSD1 [12,13,33]. p38 MAPK regulates mRNA stability and the activity of C/EBPβ by phosphorylation [34,35]. Here, IL-1α/TNFα treatment increased Thr235 phosphorylation of C/EBPβ and this was attenuated by p38 MAPK inhibition, as was the pro-inflammatory cytokine up-regulation of C/EBPβ mRNA levels. This occurred concurrently with decreased *HSD11B1* mRNA levels, suggesting that a p38 MAPK-C/EBPβ pathway mediates this process. MEK inhibition increased *HSD11B1* mRNA levels, though whether this was via MEK or ERK (ERK is activated by MEK phosphorylation) was not investigated. The relevance of this remains unknown but may reflect stabilisation of the short-lived C/EBPβ or another factor required for *HSD11B1* transcription.

As for myriad other stimuli, pro-inflammatory cytokine induction of *HSD11B1* requires C/EBPβ to bind to the FP3/4 region of the P2 promoter, and phosphorylation of C/EBPβ at Thr235 is implicated in the cytokine-induced up-regulation of *HSD11B1*. Ectopic expression of C/EBPβ mutants in transfected A549 cells provides direct evidence that Thr235 phosphorylation is critical for the IL-1α induction of *HSD11B1*. Indeed, abolition of phosphorylation at the equivalent threonine of chicken C/EBPβ by mutation to alanine (T220A), attenuated cytokine induction of the *HSD11B1* promoter, whilst ectopic expression of constitutively active C/EBPβ (mutation of Thr220 to aspartate) increased basal activity of the *HSD11B1* promoter but did not affect the level of cytokine-induced *HSD11B1* mRNA, which remained similar to that conferred by wild type C/EBPβ. However, other post-translational modifications of C/EBPβ are also likely to play a role in pro-inflammatory cytokine induction of *HSD11B1* as mutation of threonine 220 to alanine did not totally abolish the effect of IL-1α, though we cannot rule out an effect of endogenous non-mutated C/EBPβ.

Thus, p38 MAPK, C/EBPβ and NF-κB/RelA are required for the full induction of *HSD11B1* in human A549 epithelial cells by pro-inflammatory cytokines and their relative importance may differ according to cell type and activating stimulus.
